# Increased colonic propionate reduces anticipatory reward responses in the human striatum to high-energy foods^1^,^2^,^3^

**DOI:** 10.3945/ajcn.115.126706

**Published:** 2016-05-11

**Authors:** Claire S Byrne, Edward S Chambers, Habeeb Alhabeeb, Navpreet Chhina, Douglas J Morrison, Tom Preston, Catriona Tedford, Julie Fitzpatrick, Cherag Irani, Albert Busza, Isabel Garcia-Perez, Sofia Fountana, Elaine Holmes, Anthony P Goldstone, Gary S Frost

**Affiliations:** 4 Nutrition and Dietetic Research Group, Division of Diabetes, Endocrinology and Metabolism, Faculty of Medicine,; 5 Computational, Cognitive and Clinical Neuroimaging Laboratory and; 6 Centre for Neuropsychopharmacology, Division of Brain Sciences, and; 7 Clinical Imaging Facility, Imperial College London, Hammersmith Hospital, London, United Kingdom;; 8 Stable Isotope Biochemistry Laboratory, Scottish Universities Environmental Research Centre, University of Glasgow, Glasgow, United Kingdom;; 9 School of Science, University of West Scotland, Hamilton, United Kingdom; and; 10 Department of Surgery and Cancer, Computational and Systems Medicine, Imperial College London, South Kensington Campus, London, United Kingdom

**Keywords:** propionate, striatum, reward, fMRI, appetite

## Abstract

**Background:** Short-chain fatty acids (SCFAs), metabolites produced through the microbial fermentation of nondigestible dietary components, have key roles in energy homeostasis. Animal research suggests that colon-derived SCFAs modulate feeding behavior via central mechanisms. In humans, increased colonic production of the SCFA propionate acutely reduces energy intake. However, evidence of an effect of colonic propionate on the human brain or reward-based eating behavior is currently unavailable.

**Objectives:** We investigated the effect of increased colonic propionate production on brain anticipatory reward responses during food picture evaluation. We hypothesized that elevated colonic propionate would reduce both reward responses and ad libitum energy intake via stimulation of anorexigenic gut hormone secretion.

**Design:** In a randomized crossover design, 20 healthy nonobese men completed a functional magnetic resonance imaging (fMRI) food picture evaluation task after consumption of control inulin or inulin-propionate ester, a unique dietary compound that selectively augments colonic propionate production. The blood oxygen level–dependent (BOLD) signal was measured in a priori brain regions involved in reward processing, including the caudate, nucleus accumbens, amygdala, anterior insula, and orbitofrontal cortex (*n* = 18 had analyzable fMRI data).

**Results:** Increasing colonic propionate production reduced BOLD signal during food picture evaluation in the caudate and nucleus accumbens. In the caudate, the reduction in BOLD signal was driven specifically by a lowering of the response to high-energy food. These central effects were partnered with a decrease in subjective appeal of high-energy food pictures and reduced energy intake during an ad libitum meal. These observations were not related to changes in blood peptide YY (PYY), glucagon-like peptide 1 (GLP-1), glucose, or insulin concentrations.

**Conclusion:** Our results suggest that colonic propionate production may play an important role in attenuating reward-based eating behavior via striatal pathways, independent of changes in plasma PYY and GLP-1. This trial was registered at clinicaltrials.gov as NCT00750438.

See corresponding editorial on page 1.

## INTRODUCTION

Peripheral signals communicate information about current energy balance to the brain to maintain energy homeostasis (1). The hedonic properties and constant availability of highly palatable energy-dense foods promote their overconsumption and weight gain, whereas hedonic and reward-based eating behaviors are in turn influenced by peripheral homeostatic signals such as gut hormones (2–4). Hedonic responses to food are thought to involve a network of corticolimbic brain structures, and are modulated by emotional and cognitive factors, as well as sensory cues and anticipated reward.

There is increasing evidence that metabolites produced by the colonic microbiota may affect central appetite regulation (5–7). Resistant starch (RS)^12^ supplementation alters activation in hypothalamic nuclei and gene expression of neuropeptides involved in appetite regulation in rodents (5, 6). The consumption of nondigestible carbohydrates (NDCs) also reduces energy intake and weight gain in animal models (8–10). Several physiologic benefits associated with the consumption of RS and other NDCs may be mediated through the actions of their fermentation products, namely, short-chain fatty acids (SCFAs). The principal SCFAs produced via bacterial fermentation are acetate, propionate, and butyrate, present in the colon in the approximate molar ratio of 60:20:20 (11). Our research group demonstrated that increasing circulating acetate directly suppresses appetite via central hypothalamic mechanisms in rodents (7). Our recent findings from human studies suggest that propionate may also be an important SCFA contributing to appetite regulation (12). The acute intake of an inulin-propionate ester (IPE), which selectively increases colonic propionate production, reduced ad libitum energy intake and increased plasma concentrations of the anorexigenic gut hormones glucagon-like peptide 1 (GLP-1) and peptide YY (PYY), supporting results of in vitro experiments (12, 13). Furthermore, a long-term elevation in colonic propionate production protected against weight gain and reduced hepatic lipid content (12).

**FIGURE 1:**
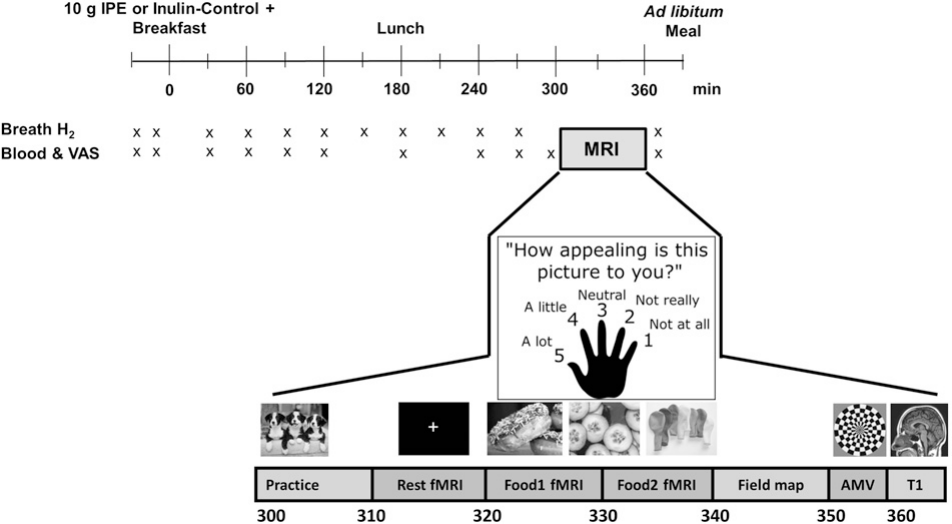
Study day protocol. Overview of timings of blood sampling, VAS ratings, breath hydrogen recordings, and scanning protocol. AMV, auditory–motor–visual; IPE, inulin-propionate ester; T1, T1 anatomical scan; VAS, visual analog scale. Adapted from reference 17 with permission.

The exogenous administration of GLP-1 or its analogs and/or PYY reduces brain reward system responses to viewing food pictures in humans (4, 14). However, to date, to our knowledge, there are no studies demonstrating an effect of SCFAs on human brain food-reward responses to influence eating behavior. In the present study, we examined the effect of an acute increase in colonic propionate production on energy intake and brain regions involved with reward processing and hedonic eating, including caudate, nucleus accumbens, amygdala, anterior insula, and orbitofrontal cortex (OFC) (15, 16), in healthy nonobese men in a randomized crossover design (**Figure 1**). We used fMRI to measure activation by the BOLD signal in these regions of interest during an established food evaluation task that used high energy (HE)– and low energy (LE)–density food pictures (primary outcome measure) (3, 17–19). We hypothesized that increasing colonic propionate production after intake of IPE would reduce anticipatory reward responses during evaluation of food pictures, a measure of food cue reactivity, compared with control inulin via the stimulation of the anorexigenic gut hormones GLP-1 and PYY (4), and would reduce ad libitum energy intake.

## METHODS

Further details are given in **Supplemental Methods**. The study was approved by the West London Research Ethics Committee (08/H0707/99) (NCT00750438).

### Participants

Subjects were recruited via public advertisement and a healthy volunteer database. Healthy men aged 18–65 y with BMI (in kg/m^2^) 20–35 were eligible for inclusion. Exclusion criteria included the following: weight gain or loss >3 kg in the previous 3 mo, any chronic illness or gastrointestinal disorder, history of drug or alcohol abuse in the previous 2 y, use of antibiotics or medications likely to interfere with energy homeostasis in the previous 3 mo, claustrophobia, contraindications for MRI scanning, daily smoking, gluten or lactose intolerance, consumption of a vegan or vegetarian diet, or depression as assessed by a Beck Depression Inventory II score >10 (20).

### Food supplements

IPE designed for targeted delivery of propionate to the colon was produced as previously described (12). Inulin was chosen as a control supplement, with the use of the same inulin used to prepare both the IPE and control supplements. This controlled for residual fermentation of the backbone NDC. In vitro fermentations of IPE and inulin suggest comparable increases in acetate and butyrate production; thus, any differences in our outcome measures can be attributed to the preferential increase in propionate production with IPE (12).

### Study day protocol

Twenty healthy men participated in this randomized, placebo-controlled, within-subject, single-blind crossover study. Subjects attended 2 separate study visits ≥6 d apart after an overnight fast. Subjects were asked to record their dietary intake; avoid caffeine, alcohol, and strenuous exercise for 24 h before each visit; and not smoke cigarettes for ≥48 h before each visit.

Study visits were conducted between April and December 2014 in the National Institute for Health Research/Wellcome Trust Imperial Clinical Research Facility, Hammersmith Hospital, London, United Kingdom. Weight, height, and body fat measurements were collected with the use of bioimpedance analysis (BC-418 analyzer; Tanita UK). At each visit, subjects completed a Positive and Negative Affect Schedule to measure mood during the previous week (21). Serial venous blood samples were collected via a peripheral cannula to assay plasma and serum metabolite and hormone concentrations over study visits (Figure 1).

Breath hydrogen concentration, a marker of colonic fermentation (22), was measured with the use of a handheld breath hydrogen analyzer (EC60 Gastrolyser Breath Hydrogen Monitor; Bedfont Scientific), and twelve 100-mm visual analog scales were completed to assess serial subjective appetite and mood ratings (Figure 1).

At 0 min, a standard breakfast containing 10 g IPE (treatment) or 10 g inulin (control) was provided to subjects in a randomized order (via sealed envelope). Breakfast was a chocolate milkshake and snack bar (574.5 kcal; 86.4 g carbohydrate, 18.8 g fat, 14.7 g protein, and 3.2 g fiber). Lunch (180 min) was a cheese sandwich and snack bar (558 kcal; 62.3 g carbohydrate, 24.9 g fat, 21.7 g protein, and 2.8 g fiber).

At 300 min, subjects completed a 60-min MRI session (Siemens 3T Verio MRI scanner) in the Imperial Clinical Imaging Facility. This time point was chosen based on previous results from an acute study that suggest successful delivery of IPE to the colon and increased plasma gut hormone concentrations after 240 min (12).

Finally, a savory meal of tomato and mozzarella pasta bake (per 100 g: 129 kcal; 17.0 g carbohydrate, 3.9 g fat, 4.8 g protein, and 3.4 g fiber) was served to subjects. Subjects were instructed to eat until they felt comfortably full. Five of the first 12 subjects who completed the study consumed all of the food presented at the meal. As a result, data on food intake for these 5 subjects were removed from analysis of ad libitum food consumption and the amount of food presented to the final 8 subjects was increased.

### fMRI scanning protocol

All subjects underwent an MRI scan from 300 to 360 min as previously described (17–19). After an initial practice run with the use of pictures of animals, subjects had a resting-state fMRI scan lasting 10 min followed by the food picture fMRI paradigm at 320 min (Figure 1). Subjects had an auditory–motor–visual (AMV) fMRI task at 350 min, followed by collection of structural magnetic resonance brain scans, including high-resolution T1-weighted scans for image registration (Figure 1). Whole-brain fMRI data were acquired with T2*-weighted gradient-echo echoplanar imaging (EPI).

### Food evaluation fMRI paradigm

During the fMRI food picture paradigm, 4 types of color photographs were presented in a block design (6 pictures/block; each image displayed for 2500 ms) split across 2 runs as follows: *1*) 60 HE foods (e.g., pizza, cakes, and chocolate), *2*) 60 LE foods (e.g., salads, vegetables, and fish), *3*) 60 non–food-related household objects (e.g., furniture and clothing), and *4*) 180 blurred images of the other pictures (as a low-level baseline) in blocks after every food or object block (17–19). While each image was on display in the scanner, subjects were asked to rate simultaneously how appealing each picture was to them with the use of a 5-button hand-held keypad (1 = not at all; 5 = a lot). Exclusion of subjects with a failure to rate >10% of the food and object pictures at either study visit was a predefined cutoff to ensure exclusion of data for subjects who may not be attending to the task.

### AMV control fMRI paradigm

An AMV control task was performed to exclude nonspecific changes in the BOLD signal between visits, as previously described (17–19). In a block design, subjects performed 2 of each of the following tasks simultaneously: *1*) listening to a story, *2*) tapping their right index finger once every second, or *3*) watching a 4-Hz color-flashing checkerboard.

### Image processing

fMRI data processing was carried out with the use of FEAT version 6.00, part of FSL (Functional Magnetic Resonance Imaging of the Brain (FMRIB) software library; www.fmrib.ox.ac.uk/fsl), including field map–based EPI unwarping and temporal derivative and motion variables as covariates in the general linear model, boundary-based registration of EPI to high resolution structural space, and nonlinear registration to standard space. Higher-level analysis used a fixed-effect model to combine the 2 runs to determine activation for the following contrasts: HE food > object, LE food > object, or any food (HE or LE) compared with objects. Similar analysis was performed for the single-run AMV paradigm including the onsets of each task (auditory, motor, and visual) to contrast activation during performance of each task with that when it was not being performed.

### Whole-brain analysis

Whole-brain analysis was performed separately with the use of FEAT v6.00 for the HE and LE contrasts with the use of a paired *t* test to identify regions with significant differences in the BOLD signal between control inulin and IPE treatments with the use of both a voxel-wise correction false discovery rate, *P* < 0.05, and a cluster-wise correction family-wise error, *Z* > 2.3, *P* < 0.05.

### fMRI regions of interest

Functional regions of interest (fROIs) were determined from average group activation in a separate cohort of 21 nonobese healthy subjects from a previous study (17) for any food (HE or LE) > object in the nucleus accumbens, amygdala, insula (anterior), caudate, and OFC brain regions (**Supplemental Figure 1** and **Supplemental Table 1**). Similar fROIs were made for the AMV control task as follows: superior temporal gyrus posterior division for the secondary auditory cortex; precentral gyrus for the primary motor cortex; and lingual gyrus for the primary visual cortex (**Supplemental Figure 2**). An anatomic region of interest for the hypothalamus was also generated with the use of the mean of all anatomical T1 scans for the subjects in the current study.

### Comparison of fMRI activation between groups

The mean bilateral BOLD signal within each a priori fROI was then extracted for each individual subject for the HE and LE contrasts at each visit to measure differences between treatments. Similar analysis was performed to compare activation in the relevant fROIs between treatments in the AMV task.

### Composite appetite score

A composite score was calculated with the use of the following formula (23): [hunger + (100 – fullness) + desire to eat + appetite for meal] ÷ 4.

### Blood sample preparation

Ten milliliters of blood was collected at each time point for assay of plasma glucose (EDTA), serum insulin, and plasma gut hormones (5 mL in lithium heparin tube containing 100 μL aprotinin protease inhibitor; Nordic Pharma UK). All tubes were centrifuged at 2590 × *g* for 10 min at 4°C. Samples were separated and frozen at −20°C until analysis.

### Metabolic and hormone analysis

Glucose analysis was performed at the Department of Biochemistry, Hammersmith Hospital, with the use of a ci8200 analyzer enzymatic method (Abbott Diagnostics). A human insulin radioimmunoassay kit (Millipore) was used for insulin analysis according to manufacturer’s guidelines with 50 μL serum. PYY and GLP-1 were measured with the use of previously established in-house specific and sensitive radioimmunoassay (24, 25). SCFAs were measured at the Department of Cancer and Surgery with the use of an Agilent 7000C Triple Quadrupole GC/MS System according to a previously published method (26). Values are expressed as means ± SEMs.

## RESULTS

### Participants

Subject characteristics are given in **Table 1**. Two of the 20 men were removed from fMRI analysis because of poor compliance with the picture evaluation task (predefined as failure to rate overall >10% of the food and object pictures during either study visit), leaving 18 subjects with data for fMRI analysis.

**TABLE 1:** Subject characteristics^1^

	All subjects	fMRI analysis
Male	20 (100)	18 (100)
European Caucasian	18 (90)	17 (94)
Age, y	52 (26, 61)	55 (27, 61)
Weight, kg	79.0 ± 1.5	78.5 ± 1.5
BMI, kg/m^2^	25.2 ± 0.5	24.9 ± 0.5
Body fat, %	20.6 ± 1.1	20.6 ± 1.1
BDI-II^2^ (maximum score 63)	1 (0, 3.3)	1 (0, 3.8)
Time between visits, d	7 (7, 17)	7 (7, 13.3)

1 Values are means ± SEMs, medians (IQRs), or *n* (%). Age and anthropometric data are means of first and second study visit measurements.

2 BDI-II, Beck Depression Inventory II.

### Breath hydrogen

**FIGURE 2:**
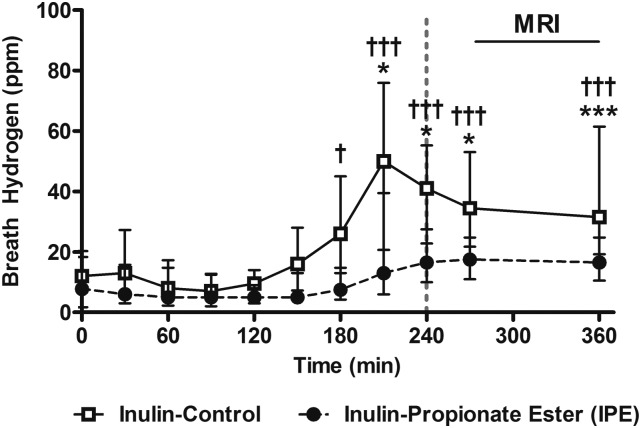
Breath hydrogen concentrations after IPE or control inulin. Values are medians (IQRs), *n* = 20. The dotted vertical line signifies the time point after which >80% IPE previously has been shown to enter the colon (12). Breath hydrogen concentrations after control inulin (†) or IPE (*) compared with baseline concentrations with the use of paired-samples *t* tests (calculations performed on normalized data): *^,†^*P* < 0.05, ***^,†††^*P* < 0.005. IPE, inulin-propionate ester; ppm, parts per million.

Breath hydrogen concentrations were significantly elevated above baseline concentrations 210 min after subjects received either inulin or IPE and stayed significantly elevated until the end of the study visit (**Figure 2**). This suggests that the fermentation of IPE and the release of propionate in the colon occurred in a time course similar to that previously reported (12). As expected, in a repeated-measures ANOVA, including treatment and time as within-subject factors, there was a significant treatment × time interaction in that breath hydrogen concentrations were significantly higher after receiving the control inulin than with IPE [*F*(1, 19) = 3.83, *P* < 0.01] because of the greater amount of fermentable carbohydrate in the control inulin than in the IPE (10 g compared with 7.3 g).

### Ad libitum energy intake

**FIGURE 3:**
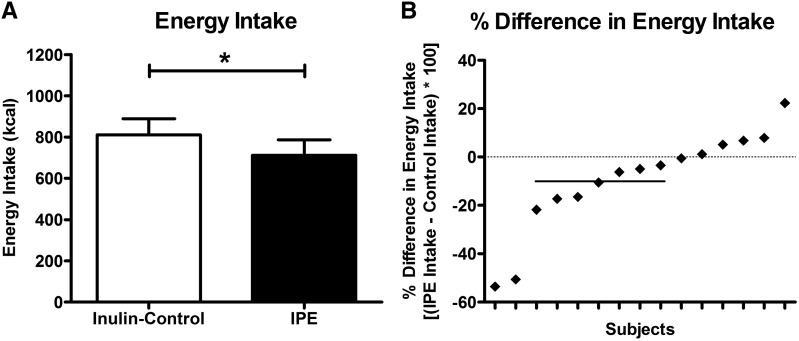
Energy intake at ad libitum meal after IPE or control inulin. Values are mean ± SEM absolute energy intake after control inulin or IPE (paired-samples *t* test: *P < 0.05, *n* = 15) (A) and individual percentage differences in energy intake between IPE and control inulin (B). The horizontal solid line in panel B represents the mean 9.5% reduction in energy intake. IPE, inulin-propionate ester.

Data on energy intake for 5 subjects were removed from analysis of ad libitum energy intake because these subjects consumed all presented food during one or both visits (see Methods). IPE treatment significantly reduced energy intake by 9.5% ± 5.3% [control 810.4 ± 83.4 kcal (95% CI: 631.6, 989.2 kcal) compared with IPE 711.1 ± 79.9 kcal (95% CI: 539.7, 882.6 kcal), *t*(14) = 2.41, *P* = 0.030] (**Figure 3**).

### BOLD signal in food evaluation fMRI task

**FIGURE 4:**
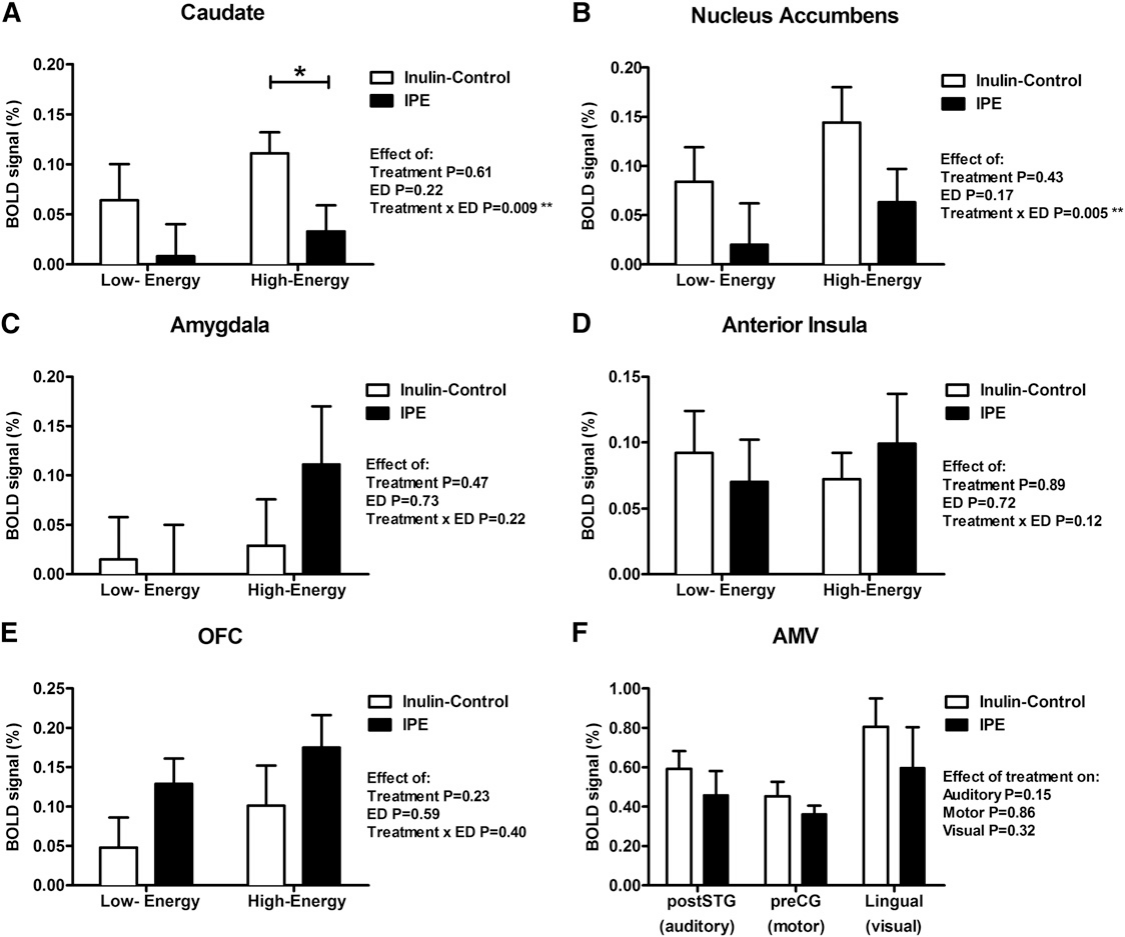
BOLD signal during food evaluation and AMV control fMRI tasks after consumption of IPE or control inulin. Magnitude of the BOLD signal (percentage) in brain reward systems in the caudate (A), nucleus accumbens (B), anterior insula (C), amygdala (D), and OFC (E) during evaluation of pictures of low-ED foods (minus objects contrast) or high-ED foods (minus objects contrast). Bilateral posterior division of superior temporal gyrus in auditory task, left precentral gyrus in motor task, and bilateral lingual gyrus in visual task after control inulin or IPE, *n* = 18 (F). Results compared with control inulin with the use of 2-factor (A–E) and 1-factor (F) repeated-measures ANCOVA with a post hoc Fisher least-significant difference test while including visit order as a covariate, *P < 0.05; **P < 0.01. AMV, auditory–motor–visual; ED, energy density; IPE, inulin-propionate ester; OFC, orbitofrontal cortex; preCG, precentral gyrus; postSTG, posterior division of superior temporal gyrus.

In 2-factor repeated-measures ANCOVA, including energy density (ED) of food pictures and treatment as within-subject factors and visit order as a covariate, there was a significant ED × treatment interaction for the BOLD signal in the caudate [*F*(1, 16) = 8.86, *P* = 0.009, Bonferroni correction *P* = 0.045 for multiple regions of interest] and nucleus accumbens [*F*(1, 16) = 10.81, *P* = 0.005, Bonferroni correction *P* = 0.025] that favored HE foods (**Figure 4**A and B), but not in the amygdala [*F*(1, 16) = 1.65, *P* = 0.22], anterior insula [*F*(1, 16) = 2.65, *P* = 0.12], or OFC [*F*(1, 16) = 0.76, *P* = 0.40] (Figure 4C–E).

In the caudate (Figure 4A), post hoc analysis revealed that IPE treatment significantly reduced the BOLD signal to HE foods [effect size: −0.078 ± 0.032 (95% CI: −0.147, −0.009), *P* = 0.029] but not to LE foods [effect size: −0.057 ± 0.037 (95% CI: −0.134, 0.021), *P* = 0.14]. However, in post hoc analysis in the nucleus accumbens (Figure 4B), IPE treatment did not significantly reduce the BOLD signal to HE foods [effect size: −0.082 ± 0.055 (95% CI: −0.198, 0.035), *P* = 0.16] or to LE foods [effect size: −0.064 ± 0.038 (95% CI: −0.144, 0.016), *P* = 0.11], although the direction of the IPE effect was similar to that for the caudate.

Independent of ED, there was no significant effect of treatment on the BOLD signal in the amygdala [*F*(1, 16) = 0.54, *P* = 0.47], anterior insula [*F*(1, 16) = 0.02, *P* = 0.89], or OFC [*F*(1, 16) = 1.56, *P* = 0.23] (Figure 4C–E).

There was no significant correlation between the difference in the BOLD signal to HE foods alone, or any food (HE or LE), in the caudate (*r* = −0.11, *P* = 0.74; and *r* = −0.41, *P* = 0.18, respectively) or nucleus accumbens (*r* = −0.19, *P* = 0.56; and *r* = −0.35, *P* = 0.27, respectively) between treatments and the difference in energy intake between treatments (*n* = 13).

In 2-factor repeated-measures ANCOVA, including ED of food pictures and treatment as within-subject factors and visit order as a covariate, there was no significant ED × treatment interaction for the BOLD signal in the hypothalamus anatomic region of interest [*F*(1, 16) = 1.61, *P* = 0.22] (**Supplemental Figure 3**). Independent of ED, there was also no significant effect of treatment on the BOLD signal in the hypothalamus [*F*(1, 16) = 1.16, *P* = 0.30].

In whole-brain analysis, there were no significant regional differences in the BOLD signal for either the HE or LE food contrasts surviving correction for multiple comparisons with the use of a voxel-wise false discovery rate of *P* < 0.05 or a cluster-wise family-wise error of Z > 2.3, *P* < 0.05.

### BOLD signal in control fMRI task

An AMV control task was performed to look for nonspecific changes in the BOLD signal between treatments, as previously described (17–19). There was no significant difference in the BOLD signal in any fROI between treatments during the control fMRI task (1-factor repeated-measures ANCOVA including visit order as covariate, *P* = 0.15–0.86; Figure 4F).

### Food appeal ratings and reaction time

**FIGURE 5:**
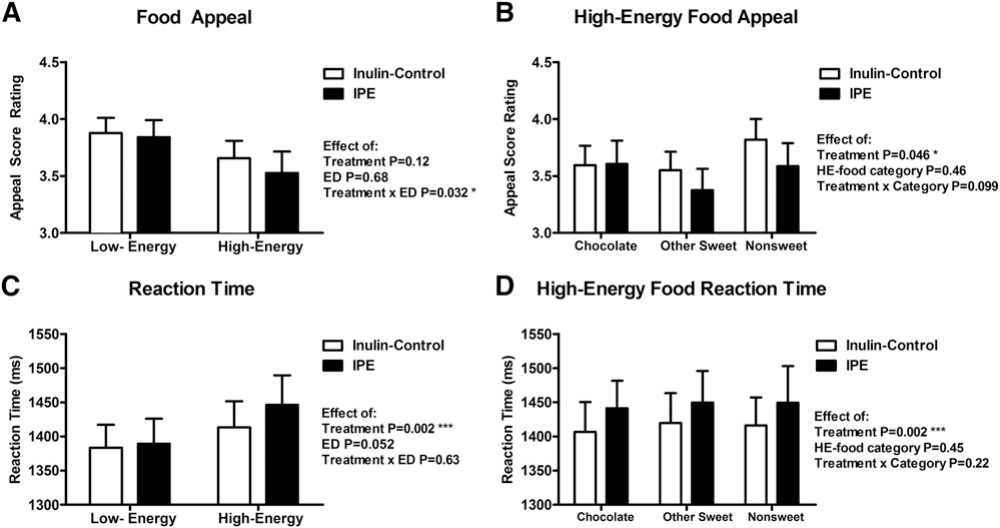
Food picture appeal ratings and rating reaction times after consumption of IPE or control inulin. Magnitude of appeal ratings (1 = not at all; 5 = a lot) (A and B) and reaction times to rate food pictures of varying energy densities as scored with the use of a hand-held button box (C and D) after control inulin or IPE (*n* = 18). Results given for low-energy or HE foods (A and C) and different categories of HE foods (chocolate, other sweet, and nonsweet savory) (B and D). Results compared with control inulin with the use of 2-factor repeated-measures ANCOVA with post hoc Fisher least-significant difference test while including visit order as a covariate, *P < 0.05; ***P < 0.005. ED, energy density; HE, high-energy; IPE, inulin-propionate ester.

In 2-factor repeated-measures ANCOVA, including ED of food pictures and treatment as within-subject factors and visit order as a covariate, there was a significant ED × treatment interaction for food appeal ratings, with a greater effect for HE foods [*F*(1, 16) = 5.50, *P* = 0.032] (**Figure 5A**). Within HE food subcategories, there was no significant HE food subcategory × treatment interaction for food appeal ratings [*F*(2, 16) = 2.49, *P* = 0.099] (Figure 5B). However, independent of HE food subcategory, HE foods were rated significantly less appealing when patients received IPE than when they received the control [*F*(1, 16) = 4.69, *P* = 0.046] (Figure 5B). By contrast, there was no difference in appeal ratings of object pictures between treatments when including visit order as a covariate [*F*(1, 16) = 0.02, *P* = 0.88].

There was no significant ED × treatment interaction for the reaction time for subjects to rate the food pictures [*F*(1, 16) = 0.24, *P* = 0.63] (Figure 5C). However, independent of ED of food pictures, IPE treatment significantly increased the reaction time to food pictures [*F*(1, 16) = 13.82, *P* = 0.002] (Figure 5C). Within HE food subcategories, there was no significant HE food subcategory × treatment interaction for reaction time to food pictures [*F*(2, 16) = 1.57, *P* = 0.22] (Figure 5D). However, independent of HE food subcategory, the reaction time for HE food pictures was significantly increased after subjects received IPE [*F*(1, 16) = 14.54, *P* = 0.002] (Figure 5D). By contrast, there was no difference in reaction times for subjects to rate object pictures between treatments when including visit order as a covariate [*F*(1, 16) = 2.40] (*P* = 0.14).

### Blood hormones and metabolites

There was no significant difference in the AUC_0–360 min_ for plasma PYY, GLP-1, or glucose or serum insulin after subjects received IPE compared with control inulin (**Supplemental Figure 4** and** Supplemental Table 2**). Furthermore, 2-factor repeated-measures ANOVA revealed no time × treatment interactions for plasma PYY, GLP-1, or glucose or serum insulin (*P* = 0.25–0.58).

### Serum SCFAs

Compared with baseline, there was no significant increase in serum acetate or propionate concentrations 240 min after subjects received IPE or control inulin (**Table 2**). For serum butyrate, there was a significant increase in concentrations 240 min after subjects received both IPE and control inulin compared with baseline. There was no difference in SCFA concentrations between treatments at baseline (*P* = 0.25–0.87) or at 240 min (*P* = 0.10–0.88).

**TABLE 2:** Serum SCFA concentrations after consumption of IPE or control inulin^1^

	μmol/L									
	Control inulin				IPE				Δ_240 min_^2^	
	0 min	240 min	*t*	*P*^3^	0 min	240 min	*t*	*P*^4^	*t*	*P*^4^
Acetate	83.9 ± 2.8	96.8 ± 7.8	−1.72	0.10	86.4 ± 4.1	95.4 ± 7.2	−1.19	0.25	0.49	0.63
Propionate	6.8 ± 0.3	7.8 ± 0.5	−1.64	0.12	7.4 ± 0.3	8.3 ± 0.5	−1.57	0.13	0.16	0.87
Butyrate	5.4 ± 0.2	6.1 ± 0.3	−2.50	0.02	5.3 ± 0.2	6.9 ± 0.4	−3.64	<0.01	−1.87	0.08

1 Values are means ± SEMs. *n* = 20. IPE, inulin-propionate ester; SCFA, short-chain fatty acid.

2 Represents concentrations at 240 min minus baseline values.

3 For 0 min compared with 240 min calculated with the use of paired-samples *t* tests within each treatment.

4 For Δ_240 min_ concentrations after control inulin compared with IPE calculated with the use of paired-samples *t* tests between treatments.

### Confounding variables

There was no significant effect of IPE treatment on composite appetite visual analog scale ratings (**Supplemental Figure 5**). There were no significant differences between treatment visits in potential confounding variables that may have affected the BOLD signal or hedonic response to food pictures, including in BMI, percentage body fat, mood, total energy intake on the previous day, ratings of nausea, sleepiness, stress or anxiety, or head motion during the fMRI task (**Supplemental Table 3**).

### Correlations between outcome measures

As expected, the change in energy intake between treatments was positively correlated with the change in composite appetite visual analog scale ratings between treatments (**Supplemental Table 4**). However, there was no significant correlation between elevated colonic propionate on the BOLD signal to HE food in the caudate or nucleus accumbens, or in the mean of both regions, and the effect of increased colonic propionate on HE food appeal, or in composite appetite visual analog scale ratings (Supplemental Table 4).

## DISCUSSION

We used an established fMRI food evaluation paradigm to assess the effects of elevated colonic propionate on brain responses during food picture evaluation in a priori regions of interest previously associated with reward processing and hedonic eating behavior. An acute increase in colonic propionate reduced the BOLD signal during an evaluation of food pictures in the caudate (dorsal striatum) and nucleus accumbens (ventral striatum) in nonobese men, which was greater for HE than for LE foods. Indeed, in the caudate, elevated colonic propionate specifically reduced the BOLD signal during evaluation of HE but not LE food pictures. Increased colonic propionate production also reduced the appeal of HE food pictures and prolonged the time taken to rate their appeal, an implicit measure that suggested reduced wanting (17, 27). Furthermore, this reduced activation in striatal brain reward systems was accompanied by a reduction in ad libitum energy intake. This is the first time, to our knowledge, that an acute increase in colonic propionate or any other SCFA has been shown to significantly reduce anticipatory food hedonic responses and associated BOLD signal changes in brain regions associated with reward processing in humans.

Changes in striatal the BOLD signal to food cues previously have been associated with physiologically relevant alterations in food reward processing and eating behavior (3, 28–30). In the satiated fed compared with fasted state, there is a reduction in the ventral striatum BOLD signal during evaluation of HE compared with LE food pictures, and a preferential reduction in the appeal of HE foods, when an fMRI paradigm identical to that of the current study is used (3). The reduced ventral striatum BOLD signal to HE food pictures in the fed compared with fasted state has also been correlated with a subsequent reduction in ad libitum energy intake (28). Similarly, satiation decreases BOLD responses to food taste in the nucleus accumbens (31), whereas duration of food deprivation correlates with a greater caudate BOLD signal in response to actual and anticipated receipt of palatable food (29). Satiation also decreases regional cerebral blood flow at rest, another marker of neuronal activity, in the caudate and nucleus accumbens (32). Greater activation in the nucleus accumbens to food pictures is predictive of future snack consumption and reduced weight loss success in an obesity lifestyle intervention (30, 33). Alterations in caudate activation to actual or anticipated receipt of HE palatable food have been linked to genetic variations in the dopamine receptor D2 (*DRD2*) gene, which is highly expressed in the striatum and linked to increased BMI, familial risk of obesity, and prospective weight gain (34–36). In addition, after Roux-en-Y gastric bypass (RYGB) surgery, obese patients have lower activation in the caudate and nucleus accumbens when HE foods are evaluated, and have lower HE food appeal than patients after gastric banding surgery (18). Acute suppression of plasma PYY and GLP-1 in patients after RYGB surgery increases food reward responses in the nucleus accumbens (19). RYGB has been associated with increased colonic propionate production in animal models (37). This evidence supports a role of the striatum in altered reward processing and food consumption patterns. It suggests that increased colonic propionate may modulate the engagement of this brain-reward circuit, resulting in a reduction in energy intake, although we could not demonstrate a direct correlation between changes in the BOLD signal and energy intake in our study.

Previous research in animal models supports the role of NDCs and SCFAs in central appetite regulation. RS supplementation reduces activity in hypothalamic appetite regulation centers as assessed by manganese-enhanced MRI and stimulates anorexigenic hypothalamic pro-opiomelanocortin expression in rodents (5, 6). Acetate itself suppresses appetite via central hypothalamic mechanisms (7) and also has been shown to have other central effects (38). By contrast, no known studies have investigated the effect of SCFAs on central appetite regulation in humans. We did not find any significant effect of elevated colonic propionate on the hypothalamic BOLD signal during food picture evaluation in the current study. However, BOLD imaging in the hypothalamus has poor reliability related to its small-size, partial-volume effects because of close proximity to the third ventricle and cerebrospinal fluid spaces, and artifact from the internal carotid artery. Furthermore, an interpretation of changes in the overall BOLD signal in the hypothalamus may be complicated by the multiple smaller hypothalamic nuclei containing both orexigenic and anorexigenic feeding neurons.

One of the major limitations of NDC supplementation is that large doses are needed to observe effects that are in line with those noted in animal studies. For example, our research group previously has shown that >35 g NDC/d is needed to suppress appetite and stimulate an increase in plasma PYY concentrations (39). However, the development of IPE uniquely allows for the targeted delivery of a known amount of propionate directly to the colon, which normally only would be obtained from a high-fiber diet, without the associated gastrointestinal side effects (e.g., abdominal bloating or flatulence). We have previously shown with the use of a ^13^C-labeled variant of IPE that >80% of the bound propionate is released coincident with breath hydrogen. This suggests delivery of the majority of bound propionate to the colon (12). We estimate that a 10-g dose of IPE delivers 2.36 g propionate to the colon, which is 2.5 times habitual daily propionate production. In the current study, the breath hydrogen data suggests significant fermentation by 210 min and at the time of the MRI session. Despite this, we were unable to detect any difference in serum propionate between treatments. Our current measurement may have lacked sensitivity to record minor increases in peripheral circulatory propionate, particularly when measured at a single postprandial time point. This is unsurprising, because >95% propionate present in the portal vein is extracted by the liver, resulting in only a minor fraction reaching the peripheral circulation (40). Nevertheless, we have previously demonstrated a significant increase in propionate ^13^C enrichment in the peripheral circulation, revealing that the bound propionate from IPE is absorbed from the gut and is available systemically (12). The significant increase in butyrate observed after both treatments could be explained by the interconversion of acetate to butyrate from microbial fermentation of inulin (41). However, it is unclear why this would be reflected in serum butyrate when splanchnic extraction has been shown to balance gut butyrate production (42).

In addition to reduced striatal responses to food pictures, we observed a 9.5% reduction in energy intake after subjects received IPE, in line with previous observations (12). However, there was no difference in plasma PYY and GLP-1 concentrations between treatments, a finding that is not in line with our original hypothesis. It previously has been demonstrated that an increased intake of NDCs and colonic SCFA production can improve body composition and reduce energy intake independent of changes in peripheral gut hormone concentrations in mice (6, 7, 43). This suggests that alternative mechanisms are responsible for the appetite-regulating effects associated with the consumption of NDCs and SCFAs, and for the changes in the striatal BOLD signal and food appeal during food picture evaluation in the current study. Propionate is a gluconeogenic precursor, both at the gut epithelium and liver, and ruminant studies consistently have demonstrated that an elevated portal concentration of propionate depresses energy intake, which is abolished with hepatic vagotomy or total hepatic innervation (44, 45). However, we did not identify any difference in blood glucose or insulin concentrations between treatments, in agreement with previous reports (12). Other possible mechanisms driving our observations may be via the induction of vagal signaling in the gut or portal vein through stimulation of free fatty acid receptor 3 (46–48). Further work is needed to gain a better understanding of the gut–liver–brain signaling pathways in response to increased colonic propionate. This would provide information about whether our observations are a direct effect of propionate on neural pathways or a secondary response mechanism to propionate metabolism.

We believe the major strengths of our study are the following: *1*) the use of fMRI, a well-validated noninvasive measure of human brain activity; *2*) the incorporation of a well-established food evaluation paradigm that is sensitive to peripheral signals influencing appetite and reward-based eating behavior, such as gut hormones and after bariatric surgery (17–19); *3*) the exclusion of nonspecific effects on the BOLD signal with the use of a control fMRI task; and *4*) the within-subject crossover design. A limitation of our study is the lack of energy intake data for 5 subjects, which may have reduced the ability to detect correlations between outcome measures. Another limitation was the inclusion of only a nonobese male cohort. The findings will need confirmation in an obese cohort and in women, who may have altered reward and emotional responses to food. The effect of long-term IPE supplementation on striatal reward responses also remains to be elucidated.

In conclusion, elevated colonic propionate significantly reduced the striatal BOLD signal during evaluation of HE foods, reduced HE food picture appeal, and reduced energy intake at an ad libitum meal in nonobese men, an effect that was independent of changes in plasma PYY, GLP-1, and glucose and serum insulin. These results suggest that colonic propionate may play an important role in human appetitive and reward-based eating behavior at least in part through brain striatal pathways.

## Footnotes

12 Abbreviations used: AMV, auditory–motor–visual; aROI, anatomical region of interest; *DRD2*, dopamine receptor D2; ED, energy density; EPI, echoplanar imaging; fROI, functional region of interest; GLP-1, glucagon-like peptide 1; HE, high-energy; IPE, inulin-propionate ester; LE, low-energy; NDC, nondigestible carbohydrate; OFC, orbitofrontal cortex; PYY, peptide YY; RS, resistant starch; RYGB, Roux-en-Y gastric bypass; SCFA, short-chain fatty acid.
